# Acute Appendicitis in a Pediatric Minority Population: Uncommon Presentations of a Common Disease

**DOI:** 10.7759/cureus.79948

**Published:** 2025-03-03

**Authors:** Fadi Aeed, Lubna Jammal, Amir Farah, Amir Mari, Wisam Abboud

**Affiliations:** 1 Neurology, Rambam Medical Center, Haifa, ISR; 2 Pediatrics, Bnai Zion Medical Center, Haifa, ISR; 3 Surgery, Medical College of Wisconsin, Milwaukee, USA; 4 Gastroenterology and Hepatology, Nazareth Hospital Edinburgh Medical Missionary Society (EMMS), Nazareth, ISR; 5 General Surgery, Nazareth Hospital Edinburgh Medical Missionary Society (EMMS), Nazareth, ISR

**Keywords:** appendectomy, appendicitis, diagnosis, pediatric, presentation

## Abstract

Introduction

Acute appendicitis (AA) is universally regarded as a leading cause of emergency surgery. Both a fiber-rich diet and socioeconomic status are suggested to affect the clinical and epidemiological properties of AA. The Arab minority, the largest ethnic minority in Israel, has unique dietary habits and socioeconomic characteristics, which may influence the presentation and outcomes of AA. Despite the known seasonal patterns of AA incidence, especially during summertime, variations within specific ethnic subpopulations remain underexplored.

Methods

We conducted a retrospective study focused on pediatric patients from the Arab minority in Israel. Clinical data were collected and analyzed from 401 pediatric patients with a primary diagnosis of AA. The collected variables included vital signs, laboratory workups (white blood cell (WBC) counts, C-reactive protein (CRP), and neutrophil percentages), and surgical reports. The analysis aimed to identify differences between simple and complicated AA cases within this subpopulation and compare the findings with reports from both Israel and worldwide.

Results

A total of 401 pediatric patients from the Arab minority with AA were analyzed. Male predominance was observed, with 62.5% (n = 251) of cases being males. Despite the commonly reported summertime peak for AA, our data showed no significant seasonal variability for both simple and complicated AA. Regarding clinical presentation, both sexes had similar blood pressure and body temperature at admission; however, females exhibited higher heart rates than males. Notably, over 90% of patients presented without fever, having body temperatures below 38°C, even in complicated cases. Laboratory findings revealed differences between simple and complicated AA. In simple AA, WBC and CRP levels were mildly elevated (WBC 13.4 ± 4.5 × 10³/mm³, CRP 3.3 ± 5.4 mg/dL). In contrast, complicated AA cases showed significantly elevated heart rates, WBC, neutrophil percentages, and CRP levels. Among biomarkers, WBC <10⁴ cells/mm³ effectively ruled out complicated AA (negative predictive value (NPV) 98.7%, sensitivity 98%), while CRP >5 mg/dL had the highest specificity (81%) but lower sensitivity (54%) and limited predictive value (NPV 92%). The complicated AA rate was 12.7%, which is lower than previously reported in regional and global studies. Additionally, 18.5% (n = 74) of patients received non-operative management (NOM), a higher rate than typically reported locally.

Conclusions

This study highlights key clinical patterns and diagnostic insights among AA patients from a pediatric minority population. Arab children demonstrate male predominance, consistent AA rates across seasons, and are less likely to present with fever or complicated AA compared to other populations. Furthermore, WBC and CRP emerge as useful tools for ruling out complicated AA, despite their limited predictive value for confirming it. Notably, the low complicated AA rates and higher NOM rates (18.5%) may reflect unique dietary habits and healthcare accessibility patterns within this minority group. Our findings contribute to a more nuanced understanding of AA presentation in diverse populations and underscore the importance of tailored clinical assessments for minority subpopulations.

## Introduction

Acute appendicitis (AA) is among the major causes accounting for acute abdominal pain in pediatric emergency rooms (ERs) and is the most common diagnosis leading to emergent abdominal surgery [1,2]. AA is mostly simple but can present as complicated with appendiceal perforation and abscess formation [3]. Complicated AA rates vary between publications. Recent studies performed in Israel and worldwide reported complication rates exceeding 20% [4-6]. While the mechanism of AA development is poorly understood, multiple publications suggested a higher incidence of AA in patients reporting low fiber intake [7-9].

AA diagnosis is based on a combination of clinical presentation, radiological evidence - mainly ultrasound (US) and computed tomography (CT) - and blood biomarkers. The clinical presentation classically consists of early abdominal pain that can be accompanied by tenderness, fever, anorexia, vomiting, and pain evoked by movement [10]. Fever incidence as a presenting sign varies within different age groups among pediatric patients diagnosed with AA. Generally, the incidence rates of fever are higher than 50% at presentation [11-14]. Biomarkers and laboratory measurements, such as white blood cell (WBC) count, C-reactive protein (CRP) levels, and neutrophil percentage, help clinicians to stratify the risk of having AA in general and complicated AA in particular. These biomarkers can aid in the diagnostic process, yet they are neither specific nor sensitive enough to rule in or out AA [3,14,15].

Uncomplicated AA can be managed conservatively using antibiotics and pain relievers or surgically by dissecting the appendix. While multiple randomized control trials confirm the safety and efficacy of conservative treatment, appendectomies continue to be the standard of care for uncomplicated AA [16]. In a large study including more than a hundred thousand pediatric patients with AA, the rates of non-operative treatment were 14% [17]. A recent study conducted in Israel reported approximately 6% non-operative management (NOM) in pediatric AA patients [18]. Interestingly, complications and outcomes were shown to differ within different population demographics and socioeconomic status [9,19-21]. Rates of complicated AA vary between different studies but are most often reported as 30% [22-25].

Twenty-one percent of the Israeli population is of Arab ethnicity. The Arab minority in Israel is a unique subgroup in many different aspects. From a socioeconomic perspective, the Arab minority suffers from greater poverty rates compared to the non-Arab Israeli population [26]. Moreover, there are disparities between the two groups regarding some of the general health parameters. For instance, newborn death rates (5.4 vs. 2.3 per 1000 live births) as well as lower life expectancy for males and females and less accessible healthcare services [27,28].

Given that Israeli Arabs and Middle Eastern Arab culture gravitates toward high-fiber diets [29,30], which are considered a protective factor for AA, we might also hypothesize that Israeli Arabs would experience complicated AA at lower rates. Furthermore, we aim to inquire whether the unique aspects of the Arab minority, as mentioned above, affect the clinical presentation of AA, its biomarkers, and management, compared to data reported from the general population worldwide as well as locally in Israel.

## Materials and methods

Study design and patient population

A retrospective, single center study was performed at the Nazareth Hospital Edinburgh Medical Missionary Society (EMMS), in Nazareth, Israel. Information was cumulated from the database of the “Nazareth Hospital EMMS.” The study included 401 Arab pediatric patients, under the age of 18, who were diagnosed with AA between January 2015 and December 2022. Patients above the age of 18, as well as patients with an incidental intraoperative diagnosis of appendicitis were excluded. AA was diagnosed clinically by a surgeon, as well as radiologically, using US or CT. All diagnoses were validated using hospital discharge records.

All measures, including vital signs and laboratory workups, were acquired at the first medical contact in the ER, minutes after admission. White (non-inflamed) appendix cases, patients admitted for elective surgeries, and pregnant patients were excluded. It is important to note that patients could have approached the pediatric ER without a doctor’s referral or delay. For most patients, the ER was located within a 20-minute drive.

Acquired parameters

Patients were managed either surgically, both via open or laparoscopic surgery, or by NOM using antibiotics and supportive care. For all patients, clinical data and laboratory parameters were recorded: age, gender, hospitalization period, month of admission, AA complications, WBC, neutrophils percentage, CRP levels, and management method of choice.

Data analysis, statistics, and ethical considerations

For statistical analyses, the chi-square test for categorical parameters and two-tailed t-tests for nominal parameters were used. All statistical analyses were performed using JASP software (JASP Team, University of Amsterdam, Amsterdam, Netherlands). All graphs were performed using Microsoft Excel software (Microsoft® Corp., Redmond, WA). All parameters in our study are presented as the mean ± standard deviation.

This study was conducted in accordance with the institutional review board of the Nazareth Hospital EMMS. Data were both analyzed and presented anonymously.

## Results

Patient demographics and characteristics

Our retrospective study was performed on a group of 401 pediatric patients, younger than 18 years old, who were admitted to our community-based hospital in Nazareth between January 2015 and December 2022.

Our institution serves the general population in northern Israel. However, due to its urban placement within Nazareth city, the largest Arab city in Israel, most of our patients belonged to the Arab minority who live near the hospital, not necessarily in Nazareth city (less than a 20-minute drive).

**Figure 1:**
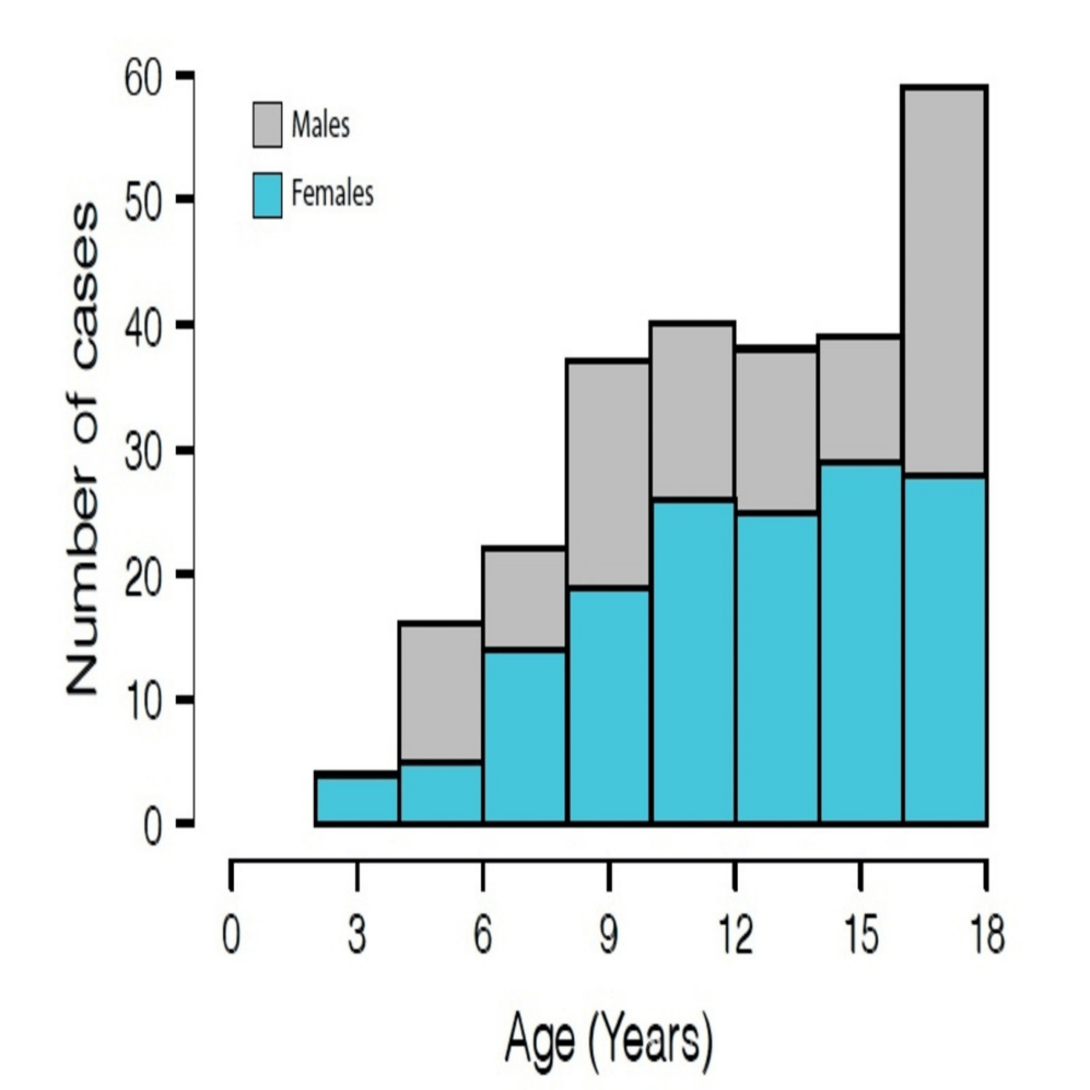
Age histogram of all acute appendicitis patients included in the study The X-axis represents the patient age in years at presentation, and the Y-axis represents the number of cases registered. Grey and bright blue represent male and female patients, respectively.

As exhibited in Table 1 and Figure 1, however, when examining age-specific incidence rates, there were certain age groups in which females had a greater incidence of AA. This distinction should be considered when interpreting the demographic trends of AA cases. The mean age for both genders was 12.8 ± 3.7, and the median was 13 years old. The mean hospitalization period was 2.6 ± 1.6 days, similar in both genders.

**Table 1:** Patient characteristics at presentation to the emergency room AA, acute appendicitis; bpm, beats per minute; NOM, non-operative management

Parameter	Value (mean ± SD or count)
Sex (male:female)	251:150 (62.5% male)
Age (mean ± SD, median)	12.8 ± 3.7 years (median: 13)
Hospitalization period (days)	2.6 ± 1.6
Complicated vs. simple AA	51:350 (12.7% complicated)
Management (NOM vs. surgical)	74:327 (18.5% NOM)
Surgical type (open vs. laparoscopic)	174:153 (53.2% laparoscopic)
Admission body temperature (°C)	37.2 ± 3.6
Body temperature >37.5°C	83 (20.9%)
Body temperature >38°C (fever)	39 (9.9%)
Admission heart rate (bpm)	99 ± 20
Systolic blood pressure (mmHg)	120 ± 14
White blood cell count (×10³/µL)	13.8 ± 4.6
Neutrophil percentage (%)	78.5 ± 11.6
C-reactive protein (mg/dL)	4.1 ± 6.5

All patients included in our study were discharged from the hospital with a diagnosis of AA. NOM was preferred in 18.5% (n = 74) of cases and 12.7% (n = 51) presented with complicated AA (defined as AA with mainly perforation, abscess formation, peritonitis, or a combination). As for the preferred surgical approach, 53% (n = 153) of patients treated surgically had an open appendectomy.

Although fever is considered in many studies as a common presenting sign in AA [11-14], our data unexpectedly exhibit the opposite, as presented in Table 1. Only the minority of cases (9.9%) presented to the ER with body temperature above 38°C. Systolic blood pressure measured at admission was 120 ± 14 mmHg, and the average heart rate was 99 ± 20. Whereas systolic blood pressure was not significantly different between sexes, heart rate at admission was significantly higher in girls (105 ± 20 vs. 96 ± 20 beats per minute (bpm)).

With regards to blood biomarkers, our patients presented with average WBC of 13.8 ± 4.6 (103 cells/mm³), 78.5 ± 11.6% neutrophil percentage, and 4.1 ± 6.5 (mg/L) CRP levels. Only 39% of patients had WBC higher than 15 (103 cells/mm³) and less than 22% had their CRP levels higher than 5 mg/dL (Table 1).

Seasonal variations in AA incidence rates

**Figure 2:**
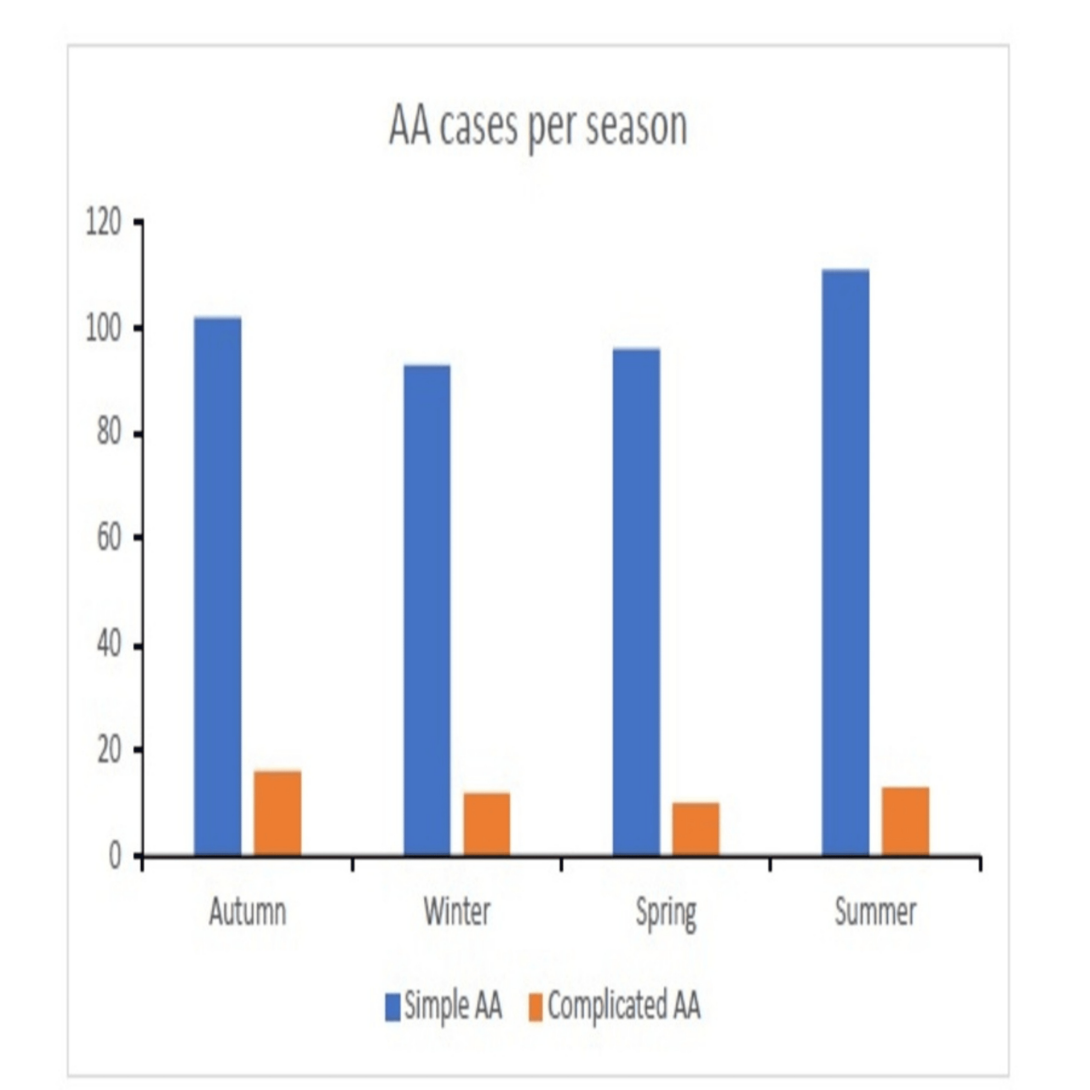
Seasonal variation in incidence rates Pediatric patients with acute appendicitis exhibit no seasonal variation. The bar plot presents the number of cases in each season: autumn (September to November), winter (December to February), spring (March to May), and summer (June to August). The Y-axis represents number of cases, and the X-axis represents the season. Blue and orange stand for simple and complicated acute appendicitis (AA), respectively.

Lines of evidence propose seasonal variation and peak incidence rates of AA during the summertime [9,31,32]. In our study, season-based analysis was conducted for simple and complicated AA cases. The absolute number of registered cases was measured for each season over the course of eight years (Figure 2). Although the summer season prevails in the absolute number of simple AA cases, our analysis shows insignificant differences in incidence rates between seasons. Similarly, no significant variations were found among seasons regarding the incidence rates of complicated AA cases.

Simple vs. complicated AA

Patients were divided into two distinct groups, simple AA and complicated AA. Complicated AA was defined as AA with mainly perforation, abscess formation, peritonitis, or a combination. We compared the different parameters, including vital signs and laboratory workups. Table 2 summarizes the results; 12.7% of cases were complicated AA. No significant differences were found regarding age, NOM percentage and open vs. laparoscopic surgical preference between the two groups. However, while male predominance was evident in simple AA, no significant difference in incidence was to be found between sexes in the complicated form.

**Table 2:** Simple vs. complicated acute appendicitis Comparison of simple and complicated acute appendicitis characteristics at presentation to the emergency room. AA, acute appendicitis; NOM, non-operative management; NS, not significant ^a^Chi-square test; ^b^t-test two tailed; ***p-value < 0.001

Parameter	Simple AA (n = 350)	Complicated AA (n = 51)	p-value	Significance
Number of cases	350	51	-	-
Male:female ratio (%)	222:128 (63.4% male)	29:22 (56.8% male)	0.365 a	NS
Age (mean ± SD, years)	12.9 ± 3.7	12.2 ± 3.7	0.26 b	NS
Hospitalization period (mean ± SD, days)	2.4 ± 1.3	4.2 ± 2.8	2.2 × 10⁻¹³ a	***
NOM vs. surgical (%)	68:282 (19.4% NOM)	6:45 (11.7% NOM)	0.18 a	NS
Open vs. laparoscopic surgery (%)	214:136 (61.1% Open)	34:17 (66.6% Open)	0.45 a	NS
Admission body temperature (°C, Mean ± SD)	37.2 ± 3.9	37.3 ± 0.6	0.98 b	NS
Systolic blood pressure (mmHg, mean ± SD)	120 ± 13	120 ± 16	0.95 b	NS
Heart rate (bpm, mean ± SD)	98.2 ± 20	109 ± 21	9.2 × 10⁻⁴ b	***
White blood cell (WBC) count (×10³/µL, mean ± SD)	13.4 ± 4.5	17.1 ± 4.6	7.7 × 10⁻⁸ b	***
WBC > 15,000/mm³ (%)	121 (34.8%)	34 (68.6%)	-	-
WBC > 10,000/mm³ (%)	269 (77.1%)	49 (98%)	-	-
Neutrophil percentage (% mean ± SD)	77.7 ± 11	84.4 ± 8	1.1 × 10⁻⁴ b	***
C-reactive protein (CRP) (mg/dL, mean ± SD)	3.3 ± 5.4	9.9 ± 9.6	3.1 × 10⁻¹¹ b	***
CRP > 5 mg/dL (%)	62 (17.7%)	27 (54.1%)	-	-
CRP > 10 mg/dL (%)	30 (8.6%)	17 (35.4%)	-	-

Unexpectedly, body temperature at admission shows no significant difference between groups. Both groups were admitted without fever with an average body temperature of less than 37.3°C and normal range systolic blood pressure. Nonetheless, with respect to vital signs at presentation, heart rate was significantly higher among the complicated AA group (109 ± 3 bpm vs. 98 ± 20, p-value < 0.001).

Multiple blood biomarkers distinguished between the two groups. WBC, neutrophil percentage, and CRP levels were significantly higher in complicated AA (Table 2). In the simple AA group, only 34% had WBC above 15 (103 cells/mm³) and 17.7% had CRP higher than 5 mg/dL, whereas in the complicated group, the ratios were 68.6% and 54.1%, respectively.

As expected, the hospitalization period was longer for the complicated AA patients (4.2 ± 0.4 vs. 2.4 ± 0.1 days, p-value < 0.001).

Next, we used cutoff values for the different blood biomarkers to test their ability in distinguishing complicated from simple AA cases (Table 3). The cutoffs used were WBC count of 10 × 10³ cells/mm³, CRP > 5 mg/dL and neutrophil percentage higher than 80%. WBC count had 98.7% negative predictive value (NPV) and 98% sensitivity but poor specificity of 22.8%. CRP levels show NPV of 92.2% and 81% specificity but poor sensitivity of 54%. Neutrophil percentage NPV was 93.2%, sensitivity of 76.4%, and low specificity of 47%. For all biomarkers, the positive predictive value was very low.

**Table 3:** Differentiating simple and complicated AA by sensitivity, specificity, PPV, and NPV AA, acute appendicitis; NPV, negative predictive value; PPV, positive predictive value

Parameter	Sensitivity	Specificity	PPV	NPV
WBC > 10,000/µL	98.0	22.8	15.6	98.7
CRP > 5 mg/dL	54.0	81.0	29.5	92.2
Neutrophil percentage > 80%	76.4	47.1	17.4	93.2

Taken together, Arab children admitted with complicated AA presented with elevated heart rates and with neither fever nor high systolic blood pressure. Moreover, patients with complicated AA had high WBC count, neutrophil percentage, and CRP at their first medical contact. 

## Discussion

This study yielded five main findings regarding AA among pediatric patients of the Arab minority in Israel. (1) While simple AA occurred more in male patients regardless of the age group, complicated AA rates were comparable between sexes. Overall, most patients diagnosed with AA were male, yet in age-specific incidence rates, there were certain age groups in which females had a greater incidence of AA. (2) Arab pediatric patients exhibit lower rates of complicated AA cases compared to studies conducted previously in Israel [33] as well as worldwide [23-25,34]. (3) Fever at presentation is uncommon in Arab pediatric patients with either simple or complicated AA. (4) WBC count at admission can effectively rule out complicated AA. (5) There are no significant seasonal variations in AA incidence rates. Cases were almost evenly distributed throughout the year with a slight increase in the summertime.

Recent articles dealing with the clinical course and epidemiology of AA suggested an important effect of consumed food types and socioeconomic status, as well as other factors such as ethnic group, seasons, environmental exposures, and medical services accessibility [35,36]. For example, Goyal et al. [21] showed that there are racial disparities in the diagnosis and treatment among different ethnicities in the US.

The results of our study show that pediatric patients belonging to the Arab minority have “atypical” clinical presentations compared to the general population in multiple aspects. First, the vital signs expected from AA patients [10] or at least complicated AA patients, such as tachycardia, changes in blood pressure, and high body temperatures, are not met.

As opposed to our results in this regard, a paper published by Gofrit and Abu-Dalu [37], who inspected Israeli pediatric patients, showed that children with complicated AA presented with higher body temperatures but no difference in WBC count compared to the normal range. Our data suggest elevated WBC as well as afebrile presentation for most patients.

Albeit belonging to a lower socioeconomic group and potentially expected to have higher rates of complications [38], Arab children exhibit lower rates of complications, lower rates of surgical interventions, and higher rates of NOM (18.5%) (Table 1). Two reasons might explain these surprising results. First, our institute is community-based and located in proximity to most patients, making the ER easily accessible. Thus, patients and their families have a higher tendency to prefer NOM over immediate surgery. Second, as mentioned above, the Arab minority in Israel consumes fiber-based food and less processed ingredients, which may play a crucial role in the lower complication rates. However, further research is needed to confirm these speculations.

For years, blood biomarkers such as WBC count and CRP levels have helped in risk stratification in AA cases. Zani et al. [39] tested the diagnostic value of the mentioned biomarkers in cases of complicated AA. Their results show that for CRP, the NPV was 80% and the PPV 38%. They also suggested similar values of neutrophil count for simple and complicated AA.

Our data in Arab pediatric patients show that from all cases with simple AA, only a small portion presented with elevated CRP levels and WBC counts. However, in complicated AA cases, WBC had a very high sensitivity and NPV (98% and 99%, respectively), making it a very useful tool to rule out complicated cases. Additionally, CRP showed high specificity of 81% and NPV of 92% (see Table 3). In addition, the neutrophil percentage was significantly higher in complicated cases.

Finally, multiple studies worldwide recognized a seasonal pattern for AA incidence, and some showed month-by-month variations [9,31,32]. These papers show that children tend to present more with AA during summertime. Although higher rates were reported in the summer, the difference was not statistically significant in our study (Figure 2).

This study has several limitations that should be acknowledged. First, its single center, retrospective design may limit the generalizability of the findings. The exclusive focus on Arab pediatric patients restricts comparisons with other ethnic groups, limiting broader applicability. Additionally, the sample size for complicated AA cases was relatively small, which may reduce the statistical power of subgroup analyses. The seasonal analysis lacked adjustments for external factors like school breaks or dietary changes, which could influence incidence patterns. Lastly, while rates of NOM were reported, the study did not assess treatment outcomes for non-operative patients, such as recurrence or treatment failure rates. Addressing these limitations in future multi-center, prospective studies with larger, more diverse populations and extended follow-up periods could provide a more comprehensive understanding of AA presentation and outcomes in this unique population.

## Conclusions

This study provides some of the first characterizations of AA presentation among the Arab minority in Israel. Given the notable cultural and socioeconomic differences between Arab and non-Arab citizens of Israel, we expected and found differences in AA presentation. Future prospective studies should aim to explain why these differences occur in similar patient populations and potentially reveal mechanisms by which AA severity can be reduced.
